# ICECREAM: randomised phase II study of cetuximab alone or in combination with irinotecan in patients with metastatic colorectal cancer with either *KRAS, NRAS, BRAF and PI3KCA* wild type, or G13D mutated tumours

**DOI:** 10.1186/s12885-016-2389-8

**Published:** 2016-05-31

**Authors:** Eva Segelov, Paul Waring, Jayesh Desai, Kate Wilson, Val Gebski, Subotheni Thavaneswaran, Elena Elez, Craig Underhill, Nick Pavlakis, Lorraine Chantrill, Louise Nott, Michael Jefford, Mustafa Khasraw, Fiona Day, Harpreet Wasan, Fortunato Ciardiello, Chris Karapetis, Warren Joubert, Guy van Hazel, Andrew Haydon, Tim Price, Sabine Tejpar, Niall Tebbutt, Jeremy Shapiro

**Affiliations:** St Vincent’s Clinical School, University of New South Wales, Sydney, NSW Australia; University of Melbourne, Melbourne, Australia; Royal Melbourne Hospital, Melbourne, Australia; Peter MacCallum Cancer Centre, Melbourne, Australia; NHMRC Clinical Trials Centre, University of Sydney, Sydney, Australia; Vall d’Hebron University Hospital, Barcelona, Spain; Border Medical Oncology, Albury-Wodonga, Australia; Northern Cancer Institute, Royal North Shore Hospital, University of Sydney, Sydney, Australia; Macarthur Cancer Therapy Centre, Campbelltown Hospital, Sydney, Australia; Kinghorn Cancer Centre, Sydney, Australia; Royal Hobart Hospital, Hobart, Australia; Andrew Love Cancer Centre, Geelong, Australia; Calvary Mater Newcastle, University of Newcastle, Newcastle, Australia; Hammersmith Hospital, London, UK; Oncologia Medica, Seconda Università degli Studi di Napoli, Naples, Italy; Flinders Medical Centre, Adelaide, Australia; Princess Alexandra Hospital, Brisbane, Australia; Sir Charles Gairdner Hospital, Perth, Australia; Alfred Hospital, Melbourne, Australia; Queen Elizabeth Hospital, Lyell McEwin Hospital, Adelaide, Australia; University Hospitals Leuven, Campus Gasthuisberg, Leuven, Belgium; Austin Hospital, Melbourne, Australia; Cabrini Hospital, Melbourne, Australia

**Keywords:** Colorectal tumours, Cetuximab, Irinotecan, Clinical trial, Tumour mutations

## Abstract

**Background:**

Patients with metastatic colorectal cancer whose disease has progressed on oxaliplatin- and irinotecan-containing regimens may benefit from EGFR-inhibiting monoclonal antibodies if they do not contain mutations in the *KRAS* gene (are “wild type”). It is unknown whether these antibodies, such as cetuximab, are more efficacious in refractory metastatic colorectal cancer as monotherapy, or in combination with irinotecan. Lack of mutation in *KRAS*, *BRAF* and *PIK3CA* predicts response to EFGR-inhibitors. The ICECREAM trial examines the question of monotherapy versus combination with chemotherapy in two groups of patients: those with a “quadruple wild type” tumour genotype (no mutations in *KRAS, NRAS, PI3KCA* or *BRAF* genes) and those with the specific *KRAS* mutation in codon G13D, for whom possibly EGFR-inhibitor efficacy may be equivalent.

**Methods and design:**

ICECREAM is a randomised, phase II, open-label, controlled trial comparing the efficacy of cetuximab alone or with irinotecan in patients with “quadruple wild type” or G13D-mutated metastatic colorectal cancer, whose disease has progressed on, or who are intolerant of oxaliplatin- and fluoropyrimidine-based chemotherapy. The primary endpoint is the 6-month progression-free survival benefit of the treatment regimen. Secondary endpoints are response rate, overall survival, and quality of life. The tertiary endpoint is prediction of outcome with further biological markers. International collaboration has facilitated recruitment in this prospective trial of treatment in these infrequently found molecular subsets of colorectal cancer.

**Discussion:**

This unique trial will yield prospective information on the efficacy of cetuximab and whether this is further enhanced with chemotherapy in two distinct populations of patients with metastatic colorectal cancer: the “quadruple wild type”, which may ‘superselect’ for tumours sensitive to EGFR-inhibition, and the rare *KRAS* G13D mutated tumours, which are also postulated to be sensitive to the drug. The focus on establishing both positive and negative predictive factors for the response to targeted therapy is an attempt to improve outcomes, reduce toxicity and contain treatment costs. Tissue and blood will yield a resource for molecular studies. Recruitment, particularly of patients with the rare G13D mutation, will demonstrate the ability for international collaboration to run prospective trials in small colorectal cancer molecular subgroups.

**Trial registration:**

Australian and New Zealand Clinical Trials Registry: ACTRN12612000901808, registered 16 August 2012.

## Background

The ICECREAM study (Irinotecan Cetuximab Evaluation and the Cetuximab Response Evaluation Among Patients with G13D Mutation) aims to provide prospective data on the optimal use of targeted therapies in patients with metastatic colorectal cancer (mCRC) whose tumours have progressed on standard chemotherapy. The trial will study two preselected patient populations in parallel, to determine whether cetuximab is optimally used as monotherapy, or in combination with an irinotecan-based regimen. In retrospective studies, the “quadruple wild type (WT) ” genotype (no mutations in *KRAS, NRAS, PI3KCA* or *BRAF* genes) appears to select responders to EFGR-inhibitors (EGFR-I) over and above that of *KRAS* exon 2 WT alone, which was until recently the extent of standard mutation testing. Similarly, retrospective data suggest that patients whose tumours harbour the specific *KRAS* G13D mutation may be sensitive to EGFR-I, in contrast to all other *KRAS* mutations. To date, no prospective trials of EGFR-I selected by tumour mutation/WT status have been undertaken.

Trial results may affect the standard of treatment for both groups of patients, in particular defining both a highly sensitive group and potentially providing the foundation for access to EGFR-I treatment for patients with *KRAS* G13D mutated mCRC. The trial was devised and instigated as an investigator initiated study in Australia, with participation of leading cancer institutes in Italy, Spain, Belgium and England.

### Rationale for evaluating the addition of irinotecan to cetuximab in WT patients

The BOND study, undertaken in patients with irinotecan-refractory mCRC demonstrated a modest progression-free survival (PFS) benefit for cetuximab in combination with irinotecan compared with cetuximab alone [1]. Whilst the benefits were modest, toxicity was increased with the combination. Also, due to being conducted in an era prior to RAS testing, as well as the lack of tissue availability, RAS testing has not been retrospectively performed on the BOND cohort. Therefore it remains unclear whether the addition of irinotecan will provide additive benefit in patients selected for *KRAS* WT tumours. The landmark EGFR-I phase III trials in refractory mCRC elected to use the EGFR monoclonal antibodies (cetuximab and panitumumab) as monotherapy [2–4]; however, clinical use in Australia and worldwide is divided equally between monotherapy and doublet therapy in the refractory setting. Therefore, the use of cetuximab alone versus combination with irinotecan remains an important, unanswered question.

### Rationale for studying “quadruple wild type” tumours

EGFR-I administration is now restricted to patients with *RAS* WT tumours, following retrospective analyses that initially demonstrated lack of mutation in *KRAS* exon 2 (codons 12 and 13) as a positive predictive marker [4, 5], with subsequent extension of predictive molecular markers to include other exons of *KRAS* as well as of *NRAS* (exons 2, 3, 4) [6–8]. Less certain, but suggestive are data showing that sensitivity to EGFR-I also depends on the WT status of *BRAF* (exon 15) and *PIK3CA* (exon 20) genes [9, 10].

### Rationale for studying EGFR-I in patients with G13D mutations

Multiple retrospective analyses, initially in refractory mCRC [11], then in 1^st^ and 2^nd^ line chemotherapy-EGFR-I combinations [12], suggested that the subgroup with the specific codon 13 mutation: G13D appear to derive benefit from cetuximab therapy to a similar extent as *KRAS* WT patients. This is also supported in a meta-analysis [13]. The KRAS exon 2 mutation c.38G > A: pGly13Asp (G13D) accounts for ~19 % of KRAS mutations, with an absolute incidence of 8 % in mCRC [11]. Preclinical studies in cell lines and xenograft models have demonstrated a response to cetuximab and blockade of the EGFR kinase pathway in the G13D mutants, but not in other (eg G12V) mutants [11]. Based on this data, the use of cetuximab for these patients harbouring the rare G13D KRAS mutation is becoming an increasingly relevant clinical predicament.

Results from the treatment of patients with G13D mutations varied in a pooled analysis of trials with panitumumab (a fully humanized EGFR-I) covering all lines of treatment of mCRC [14]. In one first line study, patients with a G13D mutation treated with panitumumab had an unfavourable overall survival, whereas patients with a G12V mutation had a favourable outcome, which was the opposite finding to cetuximab studies [6]. However, analysis of outcomes of patients with G13D tumours showed a trend to benefit from panitumumab when compared with placebo [14]. These data further support the need for prospective studies in patients with G13D tumour mutations.

The enriched group of patients being examined in the ICECREAM trial will yield valuable information regarding other features associated with response to EGFR-I, in particular the depth of response to treatment, which has been shown to be a predictor of clinical benefit and survival [15–17]. Biological substudies examining additional biomarkers in both subgroups are planned.

## Methods and design

### Study design and objectives

ICECREAM is a randomised, open-label, phase II study of cetuximab monotherapy versus the combination of cetuximab and irinotecan. The primary objective is to determine the 6-month progression free survival (PFS) benefit of cetuximab alone or in combination with irinotecan in patients with *KRAS, NRAS, BRAF* and *PIK3CKA* wild type mCRC or *KRAS* G13D mutated mCRC. PFS is defined as the time from randomisation to disease progression according to RECIST criteria, version 1.1 [18]. Secondary objectives are to determine the response rate (as complete or partial responses according to RECIST criteria, version 1.1) in patients with “quadruple wild type” or *KRAS* G13D mutated mCRC. Overall survival benefit in the two patient cohorts will be determined based on death from any cause. The study will evaluate quality of life with FACT-C (Functional Assessment of Cancer Therapy), DLQI (Dermatology Quality of Life Index Questionnaire), and FACT-EGFRI 18 (Functional Assessment of Cancer Therapy, pertaining specifically to Epidermal Growth Factor Receptor Inhibition).

Tertiary and correlative objectives are to undertake exploratory studies of biological markers as predictors of outcome. Early tumour response will be measured by assessment of tumour shrinkage on CT imaging performed at 6 weeks post first treatment, based on independent review of volumetric tumour size.

Biological substudies aim to improve understanding of the mechanisms of cetuximab and any interaction with irinotecan in wild type and G13D mutated tumours. Since the identification of new biomarkers correlating with disease activity, and the efficacy and safety of treatments are rapidly evolving, a definitive list of biomarker studies remains to be determined at the time of these studies.

### Trial organisation

The trial was developed by Australian clinicians in collaboration with key international clinicians. The Australasian Gastrointestinal Trials Group (AGITG) is the study sponsor and the trial is coordinated by the NHMRC Clinical Trials Centre (CTC) at the University of Sydney, Australia. The legal sponsor in the European Union (EU) is Hammersmith Hospital. Randomisation and data collection are performed electronically and the CTC is responsible for management of safety, ethical and regulatory reporting, central coordination of study sites and management of statistical analyses. The trial is registered on the Australian and New Zealand Clinical Trials Registry (ACTRN12612000901808).

### Statistical methods

The sample size of 100 patients is comprised of 50 patients with confirmed “quadruple wild type” status and 50 patients with a confirmed G13D tumour mutation. An intention to treat analysis will be undertaken, with the inclusion of all randomised patients.

In keeping with the randomized phase II study design, PFS was selected as the appropriate primary endpoint as this would not be influenced by subsequent cross over from monotherapy to combination treatment, which may occur at progression on study. Statistical assumptions were on the basis of retrospective data (Table 1) with a median PFS of 1.8 months in the cetuximab-alone arm versus 4.0 months for patients with KRAS G13D–mutated tumors receiving any cetuximab therapy [11]. This corresponds to a 6-month PFS rate of approximately 10 % for the monotherapy group and 35 % for the combination group. The G13D component of the study planned to recruit 25 patients per arm to match the wild-type cohort. This enabled 80 % power (α = .05) to detect an improvement from 15 to 40 % in 6-month PFS by using the Simon design for phase II trials. This magnitude of effect was chosen because we were interested in detecting a similar degree of benefit in the KRAS G13D–mutation population that was anticipated to be practice changing in patients with wild-type tumors. PFS and OS treatment effects were described by using hazard ratios (HRs) that were estimated by using Cox proportional hazards models. Completeness of the PFS was estimated by using a published equation [19]. Waterfall plots were constructed by using the biggest decrease compared with baseline in the sum of the target lesions. Patients with a decreased sum of target lesions but with new non-target lesions were set to a zero change but still qualified as having progressive disease. QoL changes over time were modeled by using generalized estimating equations. Analyses used SAS v9.3 (SAS Institute, Cary, NC).

**Table 1:** Summary of current clinical trial PFS data on which statistical calculations are based

	KRAS unknown	KRAS WT	KRAS mutant	KRAS G13D
BOND study [1] cetuximab alone vs cetuximab + irinotecan	6 m PFS = 8 % vs. 30 %	NA	NA	NA
CO.17 [4] cetuximab vs best supportive care	N/A	6 m PFS cetuximab = 30 %	6 m PFS < 5 %	
De Roock [11] cetuximab vs no cetuximab	N/A	Median PFS 4.2 m vs 1.9 m	Median PFS 1.9 m vs 1.8 m	Median PFS 4.0 m vs 1.7 m
Tepjar [12] cetuximab vs no cetuximab as 1st line treatment	Not relevant	Median PFS 9.6 m vs 7.6 m	Median PFS 6.7 m vs 8.1 m	Median PFS 7.4 m vs 6.0 m

### Planned futility analysis

As the activity of the combination is of particular interest in patients with G13D KRAS mutated tumours, Simon’s II stage design was applied to this group of patients. Futility will be declared if in the first seven eligible patients having at least six months of combination treatment, all seven patients have progressed.

### Pre-trial screening procedures and randomisation and stratification

Entry into the study is conditional on confirmation of tumour genotype by means of mutation analysis of representative samples of diagnostic tumour tissue. *KRAS* G13D status is determined by local pathology laboratories, whereas “quadruple wild type” status is determined by a central reference laboratory, the Centre for Translational Pathology, Melbourne, Australia. Archival tumour samples from the primary colorectal cancer or any metastatic site are acceptable for mutation analysis. All specimens are received as formalin fixed, paraffin embedded tissue biopsy blocks (FFPE), which upon arrival are cut into 10 μm sections by microtome and transferred onto glass slides before de-waxing and DNA extraction. An additional 4 μm slide section is stained with haematoxylin and eosin, examined by a pathologist to determine and mark the region of tumour, and then used to determine the areas of tumour to be macrodissected from unstained slides. DNA is extracted using Qiagen FFPE DNA extraction kits and successful DNA extraction is confirmed by Qubit DNA quantitation and LabChipGX DNA fragmentation assessment. For Sanger sequencing, targeted regions of interest from exon 15 of the BRAF gene, exons 9 and 20 of the PIK3CA gene, and exons 2, 3, and 4 of both KRAS and NRAS genes are amplified separately by PCR and sequenced using BigDye terminator chemistry on an ABI 3730 genetic analyser. Mutation analysis uses Mutation Surveyor software. For next generation sequencing (NGS) analysis, targeted regions of interest from exon 15 of the BRAF gene, exons 9 and 20 of the PIK3CA gene, and exons 2, 3, and 4 of both KRAS and NRAS genes are amplified and linked to barcoded NGS adapters with a two-step multiplex PCR protocol. The resulting library is sequenced on an Illumina MiSeq next generation sequencer. Mutations are detected with MiSeq Reporter software.

Written informed consent must be obtained, and patients must be randomised before starting the study treatment and planned to start within 14 days of randomisation.

Patients are centrally randomised in a 1:1 ratio and be stratified according to “quadruple wild type” or *KRAS* G13D mutational status, and treating hospital. This publication represents the current protocol version (V4).

The ICECREAM trial opened in November 2012 with a recruitment period of two years.

### Inclusion criteria

1. Males or females with histologically confirmed colorectal cancer, aged 18 years or older
2. Metastatic disease not amenable to complete resection, as determined by investigator
3. Measurable or evaluable disease, as assessed by a CT (computed tomography) scan of the chest, abdomen and pelvis according to RECIST v1.1 criteria, within 21 days prior to randomisation
4. Prior confirmation of tumour “quadruple wild type*”* status – ie.no mutations in *KRAS and NRAS* (exons 2, 3, 4), BRAF exon 15, and PIK3CA exons 9 and 20 or *KRAS G13D mutant*, by means of mutation or relevant analysis performed on representative samples of diagnostic tumour tissue
5. ECOG (Eastern Cooperative Oncology Group) performance status 0-1
6. Received and progressed on, or intolerant of all therapies listed below, where failure is defined as radiological progression during therapy, toxicity limiting further therapy or progression within 6 months of prior treatment. Previous treatment should have ceased at least 14 days prior to randomisation.
  thymidylate synthase inhibitor (e.g. 5-fluorouracil, capecitabine, raltitrexed, tegafur − uracil, S1). Thymidylate synthase inhibitors may have been given as monotherapy or in combination with oxaliplatin or irinotecan
  irinotecan-containing regimen (ie. single agent or in combination) and still able to tolerate additional irinotecan treatment)
  oxaliplatin-containing regimen OR have documented unsuitability for an oxaliplatin-containing regimen
7. Adequate haematological and renal function within 14 days prior to randomization
  Platelets ≥ 75 × 10^9^/L, Haemoglobin ≥ 80 g/L, ANC (absolute neutrophil count) ≥ 1.0 × 10^9^/L
  Serum creatinine ≤ 1.5 × institutional ULN or creatinine clearance >50 ml/min
8. Adequate liver function with total serum bilirubin ≤2.5 x ULN, and both ALT (alanine transaminase) and AST (aspartate transaminase) ≤5.0 x ULN within 14 days prior to randomization. Patients with Gilbert’s syndrome may be included as long as unconjugated bilirubin levels fall within these limits
9. Life expectancy at least 12 weeks
10. Study treatment both planned and able to start within 14 days of randomisation
11. Willing and able to comply with all study requirements, including treatment, timing and/or nature of required assessments
12. Signed, written informed consent.

### Exclusion criteria

1. Prior treatment with cetuximab or other drugs targeting the EGFR pathway, such as panitumumab, gefitinib, erlotinib
2. Severe restrictive lung disease or radiological pulmonary findings of interstitial lung disease on the baseline chest CT which, in the opinion of the investigator, represents significant pathology
3. Brain metastases that are either untreated, symptomatic, or which have not been stable for at least one month following treatment
4. History of other malignancies except where treated with curative intent AND with no current evidence of disease AND considered not to be at risk of future recurrence
5. Severe or uncontrolled cardiovascular disease (e.g. acute coronary syndromes, cardiac failure NYHA (New York Heart Association) III or IV, clinically relevant myopathy, history of myocardial infarction within the last 12 months, significant arrhythmias)
6. Concurrent illness, including severe infection that may jeopardize the ability of the patient to undergo the procedures outlined in this protocol with reasonable safety
7. Presence of any psychological, familial, sociological or geographical condition potentially hampering compliance with the study protocol and follow-up schedule, including alcohol dependence or drug abuse
8. Pregnancy, lactation, or inadequate contraception. Women must be postmenopausal, infertile, or use a reliable means of contraception. Women of childbearing potential must have a negative pregnancy test done within 7 days prior to registration. Men must have been surgically sterilised or use a (double if required) barrier method of contraception.

### Administration of study treatment

Patient will be randomised to one of two treatment arms:

- *Arm A*
  - Cetuximab monotherapy (Cetuximab 400 mg/m^2^ IVI on Day 1, followed by 250 mg/m^2^ IVI every week).
- *Arm B*
  - Cetuximab and irinotecan combination therapy (Cetuximab 400 mg/m2 IVI on Day 1, followed by 250 mg/m2 IVI every week; Irinotecan 180 mg/m^2^ IVI on Day 1 after cetuximab infusion, then every 14 days).

Body surface area (BSA) will be calculated using actual body weight. Irinotecan dose is capped at that for BSA 2.2 m^2^; and no dose capping is stipulated for cetuximab.

The starting dose of irinotecan is at the discretion of the investigator, taking into account prior irinotecan dosing and adjustments. Whilst on study, all patients will be permitted a maximum of two further reductions in irinotecan (level 1 of −20 % and level 2 of −40 %), excluding dose reductions at commencement. Cetuximab dose reductions are permitted beyond level 2 (dose level 3 of 100 mg/m^2^, and dose level 4 of 50 mg/m^2^). Treatment will continue until disease progression, unacceptable toxicity, or patient or physician request for cessation.

In Australia, for patients with *KRAS* WT colorectal cancer, cetuximab and irinotecan are approved treatments and will be supplied through the Pharmaceutical Benefits Scheme (PBS) and available through local hospital supply. For Australian patients with *KRAS* G13D mutant colorectal cancer, cetuximab will be supplied by Merck Serono Australia Pty Ltd.

In England, Italy and Spain only patients with the G13D tumour mutation will be recruited and cetuximab is supplied by Merck Serono Australia Pty Ltd. The Belgian site will recruit patients with “*KRAS* quad wild type” tumour status and the study treatments are approved for use and sourced as per local hospital supply.

### Cetuximab administration

Cetuximab therapy should be administered prior to cytotoxic chemotherapy by intravenous infusion at an initial dose of 400 mg/m^2^ (over target duration of 120 min) and further weekly infusions at a dose of 250 mg/m^2^ (over target duration of 60 min). The infusion rate must not exceed 10 mg/min. Normal (0.9 %) saline solution is used to flush the line at the end of the infusion. Close monitoring of vital signs of the patient must be checked before, during and at end of the infusion and for at least one hour after the end of the infusion. The patient must be pre-treated with an antihistamine (e.g. loratadine) and dexamethasone prior to every infusion to reduce the risk of a hypersensitivity reaction. Cetuximab must not be mixed with any other substance.

### Irinotecan administration

Irinotecan will be administered by intravenous infusion over 90 min, protected from light. Close monitoring of vital signs of the patient must be checked before, during and at end of the infusion for cholinergic symptoms (ie. early onset diarrhoea, runny nose, increased salivation, miosis, lacrimation, diaphoresis or flushing). If these symptoms develop, the infusion must be stopped, vital signs recorded and the patient reviewed according to local institutional guidelines. The patient may be pre-treated with a 5HT3-antagonist, corticosteroid and atropine.

### Dose modifications and concomitant medications

Adverse events will be graded according to National Cancer Institute Common Terminology Criteria for Adverse Events (CTCAE) version 4.0. If there is a requirement to delay treatment due to toxicity, and that toxicity can be attributed to one specific drug, then the other treatments can be continued on schedule. If irinotecan is delayed for ≥28 days, then the patient must cease irinotecan and should continue cetuximab monotherapy. Cetuximab treatment may also be delayed for a maximum of 28 days.

In general, treatment should be withheld during adverse events of severity grade 3–4, and not restarted until the adverse event has resolved to grade 0–1, at the investigator’s discretion if not otherwise indicated in the protocol. If the adverse event (not including skin toxicity) has not resolved to grade 1 after delay of treatment for 28 days, then study treatment should be discontinued. Treatment should not be delayed or modified for alopecia of any grade.

Specified dose reductions apply to all subsequent doses of the study drug. If a patient experiences several adverse events with differing recommendations, then the modification that results in the longest delay and lowest dose should be used. Dose escalations or dose re-escalations after reductions for adverse events are prohibited. All palliative and supportive care measures and medications may be administered at the Investigator’s discretion unless otherwise stated in the dose modifications.

### Treatment discontinuation

Study treatment will be permanently discontinued for documented progressive disease as per RECIST 1.1; unacceptable toxicity (as determined by the patient or treating clinician); delay of treatment for more than 28 days due to treatment-related adverse events; if the treating clinician determines that continuation of treatment is not in the patient’s best interest; for occurrence of an exclusion criterion affecting patient safety, e.g. pregnancy or psychiatric illness; failure to comply with the protocol, e.g. repeatedly failing to attend scheduled assessments, or if the patient withdraws their consent to participate in the study. Treatment after discontinuation of study treatment will be at the discretion of the patient’s clinician.

### Ethics, informed consent and safety

The study will be performed in accordance with the Declaration of Helsinki and with all applicable regulatory requirements in Australia and in the EU Directive. Institutional ethics approval is required, and patients will provide written, informed consent. Central Ethics approval was obtained by the Clinical Trials Centre (CTC) from the Sydney Local Health District Ethics Review Committee, Royal Prince Alfred Hospital Zone, Sydney, Australia.

## Discussion

Identifying biomarkers of sensitivity to anticancer agents is the key to tailoring individual therapy, to maximise benefit and reduce toxicity. *KRAS* status as a marker of sensitivity to EGFR-I was identified in a retrospective analysis of the CO.17 study [4], with the near-perfect fit of *KRAS* mutants having no benefit from this class of agents becoming adopted into standard practice without prospective verification. At the time, mutation testing of the *KRAS* gene was limited to exon 2 only. Further analysis has led to the identification of particular predictive subgroups which may have differential benefit from EGFR-I. The first group is patients with tumours harbouring the *KRAS* exon 2 G13D mutation, who in retrospective series (with small numbers only) appear to respond to EGFR-I (in trials before *KRAS* selection became an eligibility criteria), but are currently precluded from accessing such treatment. The second group are patients who perhaps should be spared from having ineffective treatment, as they seem not to respond to EFGR-I because of mutations in other exons of *KRAS* (exons 3 and 4) or in the *NRAS*, *BRAF* and *PI3KCA* genes. The superselection of responsive patients and exclusion of patients unlikely to benefit from EGFR-I needs prospective validation.

ICECREAM is an investigator-initiated, randomised, phase II trial being conducted through the Australian Gastro-intestinal Trials Group (AGITG) and coordinated by the Clinical Trials Centre (CTC) at the University of Sydney. Initially an international phase III study for the G13D mutated population was proposed, but the numbers for this subgroup were considered unachievable. Indeed, both the necessity as well as the challenge of performing prospective randomised trials in small molecularly defined subgroups such as this has been well recognised [20]. One strategy to ensure successful recruitment has been to open in key international sites within several countries, with strongly publicised and encouraged cross referral to trial sites facilitating good uptake.

The issue of whether cetuximab activity in refractory metastatic colorectal cancer is enhanced by the addition of irinotecan chemotherapy is the other important question addressed by the ICECREAM trial. There is preclinical evidence that irinotecan may enhance the efficacy of cetuximab in the G13D mutant subgroup [11] and clinical evidence, from the BOND study [1], in an unselected refractory mCRC population. Examination of the role of addition of chemotherapy in a highly selected subgroup such as the “quadruple wild type” is therefore important.

## Summary

Therefore, this prospective study addresses the efficacy of cetuximab monotherapy compared to combination with irinotecan in two molecularly defined subpopulations of patients with refractory metastatic colorectal cancer, either “quadruple wild type” tumours or *KRAS* G13D mutated tumours. Results will inform the need for larger phase III studies, as well as prove that randomised trials in rare molecular subtypes of CRC can be successfully completed using international collaborative networks.

## Abbreviations

AGITG, Australasian Gastro-Intestinal Trials Group; ALT, alanine transaminase (or alanine aminotransferase); ANC, absolute neutrophil count; AST, aspartate transaminase (or aspartate aminotransferase); BSA, body surface area; CT, computed tomography; CTC, National Health and Medical Research Council Clinical Trials Centre; DLQI, Dermatology Life Quality Index; DLQI, Dermatology Quality of Life Index Questionnaire; ECOG, Eastern Cooperative Oncology Group; EGFR, epidermal growth factor receptor; EGFRI, epidermal growth factor receptor inhibitor; FACT, Functional Assessment of Cancer Therapy; FACT-EGFRI 18, Functional Assessment of Cancer Therapy, pertaining specifically to Epidermal Growth Factor Receptor Inhibition; IV, intravenous; IVI, Intravenous infusion; mCRC, metastatic colorectal cancer; PBS, Pharmaceutical Benefits Scheme; PFS, progression-free survival; QOL, quality of life; RECIST, Response evaluation criteria in solid tumours; ULN, upper limit of normal; WT, wild type
